# Machine Learning-Based Models to Predict Drug-Induced Liver Injury (DILI) to Assist Medicinal Chemistry

**DOI:** 10.1021/acs.jmedchem.5c02888

**Published:** 2026-06-22

**Authors:** Dominga Evangelista, Elliot Nelson, Ben Tehan, Greta Bagnolini, Marinella Roberti, Giovanni Bottegoni

**Affiliations:** † Department of Pharmacy and Biotechnology, 9296 University of Bologna, 40126 Bologna, Italy; ‡ 464286 OMass Therapeutics, Oxford OX4 2GX, U.K.; § Department of Pharmacy, University of Birmingham, Edgbaston B15 2TT, Birmingham, U.K.; ∥ Department of Biomolecular Sciences, 19044 University of Urbino, Urbino 60129, Italy

## Abstract

Drug-induced liver injury (DILI) is a leading cause of drug failure and post-market withdrawals. Traditional preclinical methods fail to detect up to 40–45% of clinical hepatotoxicity cases. Computational approaches, particularly those based on machine learning and deep learning (DL), are emerging as promising tools to support medicinal chemistry and early drug discovery, though their predictive capabilities remain under active investigation. In this perspective, we review the development of DILI annotation data sets, tracing their growth from small collections to large, comprehensive resources. We also outline the evolution of computational methods, from simple descriptor-based models to advanced DL and ensemble approaches that incorporate interpretable features. Finally, we highlight recent efforts to integrate standardized causality frameworks, pharmacogenomics, and mechanistic models, aiming to connect computational advances with clinical relevance. This perspective provides valuable insight for researchers and promotes the development of more robust and consensual DILI prediction strategies.

## Graphical abstract

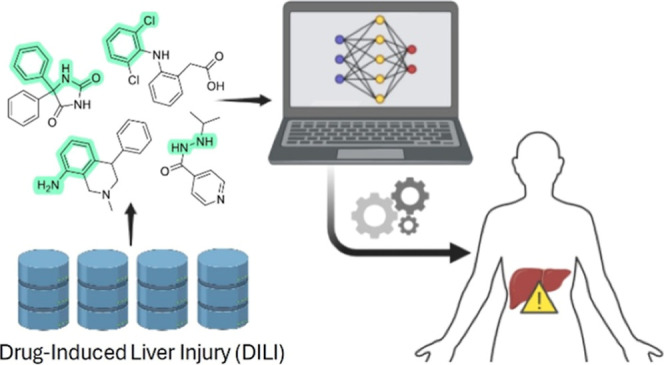

## Introduction

Drug-induced liver injury (DILI) remains a major cause of compound attrition during clinical development and a frequent contributor to post-marketing withdrawal.^1,2^ It accounts for approximately 10–15% of acute liver failure cases and continues to represent a primary safety liability in modern drug development, as confirmed by recent large-scale analyses.^1^ DILI is defined as hepatic damage from drugs/metabolites, classified as intrinsic (InDILI, dose-dependent/predictable) or idiosyncratic (IDILI, host-dependent). InDILI is typically dose-dependent and can be predicted using human or animal models. It is relatively common and usually occurs within 1 to 5 days of taking higher-than-therapeutic doses of the triggering drug. In this case, elevated hepatic exposure can result from multiple, mechanistically distinct factors. (i) Intentional or involuntary overdose, exemplified by acetaminophen at >4 g/day, which precipitates acute liver failure through excessive formation of reactive metabolites (RMs) following depletion of glutathione (GSH)-mediated detoxification pathways.^3^ (ii) Accumulation of toxic or pharmacologically active metabolites, such as desethylamiodarone derived from amiodarone, may induce phospholipidosis and steatosis; analogous effects are observed in hypervitaminosis A.^4^ (iii) Drug–drug interactions that impair metabolic clearance, for example, inhibition of CYP3A4 or CYP2C9 by ketoconazole, leading to increased systemic exposure to statins, can further elevate hepatic burden. (iv) Dosing errors represent an additional iatrogenic source of excessive exposure. (v) Reduced clearance in elderly patients or those with comorbidities may increase intrahepatic drug concentrations even at standard doses. Finally, (vi) therapeutic regimens requiring high daily doses (>100 mg) of highly lipophilic compounds (log *P* > 3) are intrinsically associated with increased hepatic exposure.^5^

InDILI typically presents as elevations in serum liver enzymes (e.g., aminotransferases or alkaline phosphatase (ALP)), often in the absence of jaundice. In contrast, idiosyncratic DILI (IDILI) is rare and inherently difficult to predict. It most commonly manifests as acute hepatocellular hepatitis, with a delayed onset ranging from 5 to 90 days after initiation of therapy. Unlike InDILI, IDILI exhibits weak dose dependence and marked interindividual variability, making it challenging to predict using animal models.^6,7^ InDILI and IDILI, despite their different clinical presentations and predictability, share common initiating mechanisms related to drug metabolism in the liver (Figure 1). In particular, they both share hepatic CYP-mediated bioactivation to RMs.^8,9^ Recurrent bioactivation pathways include quinone–imine formation, epoxidation, and N- or S-oxidation. Among 619 CYP-dependent cases, CYP3A4/5 predominates (49.6%), followed by CYP2C9 (24.6%) and CYP2E1 (13.2%). These isoforms generate reactive oxygen species [ROS] and electrophilic intermediates that promote glutathione depletion and/or immune activation.^9^

**Figure 1:**
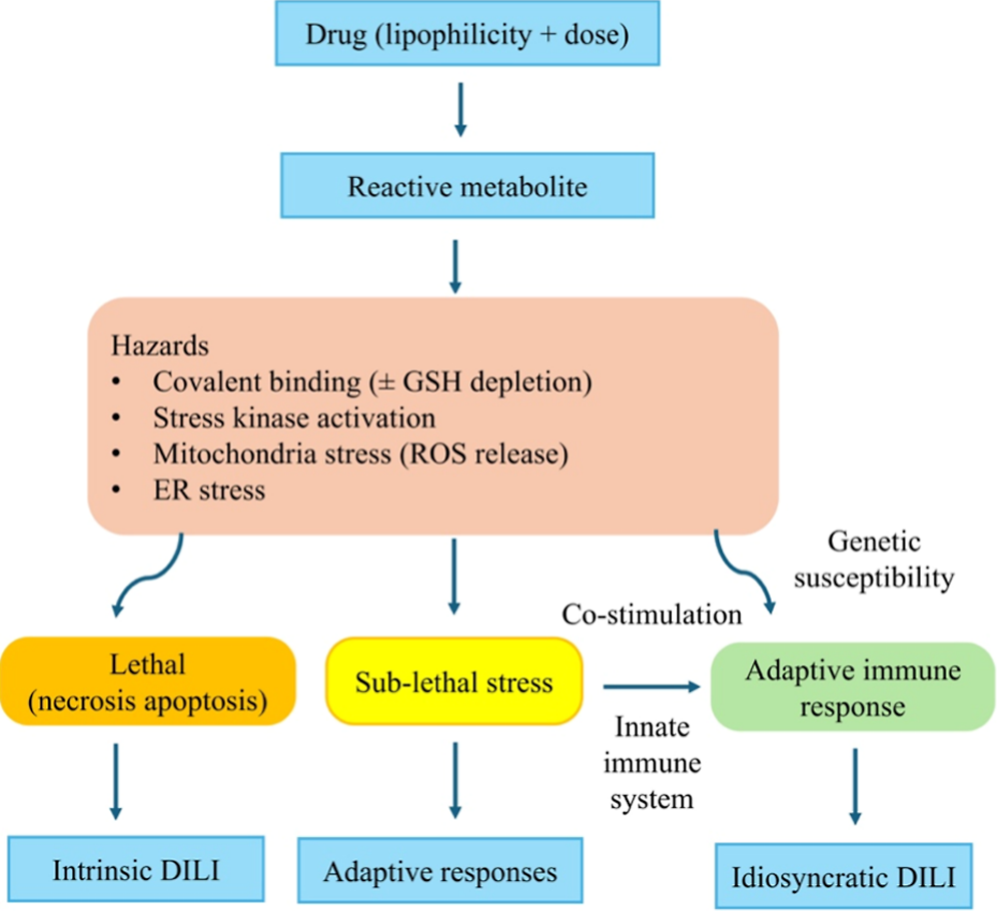
Schematic representation of the mechanistic relationship between intrinsic (InDILI) and idiosyncratic (IDILI) DILI. The diagram illustrates the common pathway starting with lipophilic drugs and their RMs, leading to cellular hazards including covalent binding (with glutathione [GSH] depletion), stress kinase activation, mitochondrial stress (with ROS release), and endoplasmic reticulum (ER) stress. The cellular response determines whether the outcome results in intrinsic DILI (through overwhelming cellular defenses) or idiosyncratic DILI (through adaptive immune responses in genetically susceptible individuals).

As illustrated in Figure 1, lipophilic drugs and their RMs are often associated with both types of hepatotoxicity. The formation of RMs can trigger several cellular hazards, including covalent binding (with glutathione depletion), stress kinase activation, mitochondrial stress with ROS release, and ER stress. The subsequent cellular response determines whether the outcome will be intrinsic DILI (through overwhelming cellular defenses leading to necrosis or apoptosis) or idiosyncratic DILI (through adaptive immune responses in genetically susceptible individuals).^10,11^ In addition to InDILI and IDILI, another important form of DILI is cholestatic liver injury. This type of liver injury occurs when bile transport is disrupted, leading to a buildup of bile acids in both the liver and bloodstream.

For several decades, hepatic biochemical indexes, namely, alanine aminotransferase (ALT), aspartate aminotransferase (AST), total bilirubin, and ALP, have been the major serum biomarkers for detecting DILI in clinics.^12^ Notably, many patients may remain asymptomatic and only exhibit elevated biomarkers after hepatocyte injury has occurred, making these biomarkers unsuitable for identifying the potential for DILI.^12^ The limitations of traditional testing methods have become increasingly apparent in recent years. Animal studies fail to identify 40–45% of liver toxicity cases that later emerge in clinical trials,^13^ highlighting a critical preclinical–clinical gap.

This translational gap is especially significant for IDILI, which arises from a rare combination of genetic and nongenetic risk factors that determine a patient’s susceptibility to a drug. The low incidence of IDILI makes it more difficult to establish a causal relationship between drug exposure and observed liver injury,^14^ reducing confidence in DILI diagnoses and making it challenging to build accurate predictive models.

To overcome these limitations, recent efforts have focused on developing comprehensive DILI annotation data sets and advanced computational approaches for toxicity prediction. In this context, machine learning (ML) approaches represent a promising alternative to traditional testing methods for DILI prediction. Indeed, ML can automatically learn patterns from diverse toxicological data and make hepatotoxicity predictions without requiring explicit programming of the biological rules. Deep learning (DL), a subset of ML employing multilayered neural networks, is particularly well-suited for DILI prediction because it can simultaneously process and integrate multiple heterogeneous data types, including molecular structures, patient demographics, genetic polymorphisms, clinical biomarkers, drug–drug interactions, and temporal patterns of liver injury progression.^15,16^ Unlike traditional approaches that typically analyze single data types in isolation, these methods excel at identifying complex interactions across diverse information sources that collectively contribute to the hepatotoxicity risk. In the context of drug discovery and development, these methods can leverage various molecular representations and biological data types to identify potential hepatotoxicity risks earlier in the drug development process. By analyzing patterns across large data sets of known hepatotoxic and non-hepatotoxic compounds, these models can potentially identify structural features and physicochemical properties associated with increased DILI risk, providing valuable guidance for medicinal chemistry optimization campaigns.

This perspective explores the evolution of DILI annotation data sets and of computational DILI prediction models, with a particular focus on ML and DL approaches that can assist medicinal chemistry efforts. We examine how these models incorporate biological information and provide interpretable predictions that can guide structural modifications to reduce the toxicity risk while maintaining therapeutic efficacy. Ultimately, we aim to highlight how these computational approaches can be integrated into drug discovery workflows to identify and mitigate DILI risk early, thereby reducing late-stage attrition and enhancing patient safety.

## DILI Data Analysis

### DILI Annotation Data Sets

Over the past two decades, large, expertly curated DILI annotation data sets have become central to both mechanistic investigation and predictive modeling. These resources consolidate physicochemical properties, clinical case reports, severity classifications, and, in some instances, mechanistic annotations and now constitute the principal training and benchmarking platforms for in silico DILI prediction models.

The following sections will examine the key DILI annotation data sets (as summarized in Table 1), focusing on how they classify DILI risk in humans, their size and scope, data collection methods, and the critical issue of standardization across different sources.

**Table 1:** Characteristics of Major DILI Annotation Data Sets^a^

data set	data type	no. of compounds	DILI end point	references
Cruz-Monteagudo	drug name	74	idiosyncratic hepatotoxicity (IH), no idiosyncratic hepatotoxicity (NIH)	*J Comput Chem* **29**: 533–549 (2008)
Xu et al.	drug name, pharmacological action, IUPAC name, canonical smiles	344	DILI positive, DILI negative	*Toxicol. Sci.* **105**, 97–105 (2008)
Greene et al.	drug name, CAS number (extension of the data set by Xu et al.)	626	HH = evidence of human hepatotoxicity (273)	*Chem. Res. Toxicol.* **23**, 1215–1222 (2010)
			NE = no evidence of hepatotoxicity in any species (152)	
			WE = weak evidence (<10 case reports) of human hepatotoxicity (62)	
			AH = evidence for animal hepatotoxicity but not tested in humans (139)	
Suzuki et al.	drug name	319	identified as causes of DILI at three major DILI registries	*Drug Saf.* **33**, 503–522 (2010)
Liew et al.	drug name, SMILES	1274	positive = with hepatic effects, (759)	*J. Comput. Aided Mol. Des.* **25**
			negative = no hepatic effects (515)	855–871 (2011)
LiverTox	ingredient; likelihood score; agent type (*P* = prescription or conventional drug; PP = prescription drug in combination or in an overview/drug class chapter; H = herbal product; I = illicit agent; M = mineral; MIDS = multi-ingredient dietary supplement; *N* = nutritional supplement)	as of 2019, approximately 1200 (1140) records were available	“likelihood score” which ranges through E (unlikely) (370), to E* (unproven but suspected) (177), D (possible) (167), C (probable) (137), B (highly likely) (104) or A (definite likelihood of causing clinically apparent liver injury) (116), X (unknown) (68)	*Hepatology* **57**, 873–874 (2013)
Zhu and Kruhlak	SMILES	282	DILI positive (177), DILI negative (105)	*Toxicology* **321**, 62–72 (2014)
FDA DILIrank	LTKB ID (LiverTox Knowledge Base identifier), PubChem_CID, LabelCompoundName, SMILES	1036	vMost-DILI concern (192), vLess-DILI concern (278), and vNo-DILI concern (312) plus Ambiguous DILI concern (254)	*Drug Discov. Today* **21**, 648–653 (2016)
FDA DILIst	drug name, routes of administration	1279	DILI positive (768), DILI negative (511)	*Drug Discov. Today* **25**, 201–208 (2020)
Proctor et al., 2017	drug name	110	1: severe clinical DILI (23)	*Arch. Toxicol.* **91**, 2849–2863 (2017)
			2: high clinical DILI concern (23)	
			3: low clinical DILI concern (23)	
			4: enzyme elevations in clinic (16)	
			5: no DILI (25)	
Vorrink et al., 2018	drug name	123	DILI positive (70 positives among them 36 severe DILI and 34 high DILI concern), DILI negative (53)	*Toxicol. Sci.* **163**, 655–665 (2018)

a The table presents key features of prominent DILI data sets including data types collected, number of compounds analyzed, DILI classification end points, and references. Data sets range from early efforts with 74 compounds (Cruz-Monteagudo) to comprehensive resources like DILIst with 1,279 drugs, showing the evolution from simple binary classifications to more nuanced categorization schemes.

Early DILI data sets, while relatively small, played an important conceptual role. Cruz-Monteagudo et al.^17^ compiled a 74-drug data set combining DILI-positive cases from a previous report by Li^18^ with manually selected, non-hepatotoxic drugs from drug compendia. In 2008, Xu et al.^19^ reported a data set of 344 drugs and chemicals with annotations derived from clinical hepatotoxicity data, drug labels, reports, and preclinical animal studies. This data set served as a foundation for an extended strong set of 626 compounds reported a few years later by Greene and colleagues.^20^ Greene’s work notably refined the classification system by introducing more nuanced categories for negative cases, distinguishing between “no evidence” and “weak evidence” of hepatotoxicity. The data set was used to evaluate the performance of a structural-alert-based DILI prediction model. Building on these efforts, Suzuki et al.^21^ compiled a 319-drug data set with broad international coverage by integrating national DILI registries from Spain, Sweden, and the USA with liver adverse-event reports from the WHO VigiBase database,^22^ whereas Zhu and Kruhlak^23^ assembled 282 compounds and, importantly, proposed a pragmatic strategy to define DILI-negative drugs based on long-term market presence without documented hepatotoxicity.

The LiverTox database represents an important resource for DILI research, providing information on liver injuries caused by drugs, dietary supplements, and herbal products. The database not only includes a collection of drug hepatotoxicity records but also incorporates results from established causality assessment methods, including the Roussel Uclaf Causality Assessment Method (RUCAM)^24^ and the Drug Induced-Liver Injury Network (DILIN) Causality Process.^25^ RUCAM employs a structured scoring system for assessing drug-induced liver injury evaluating seven criteria: time to onset, course of reaction, risk factors, concomitant drugs, alternative causes, previous information, and rechallenge response. The total score classifies causality from “highly probable” (>8) to “excluded” (≤0). For DILI research, RUCAM provides a certain degree of standardization that reduces interobserver variability. This is complemented by the DILIN Causality Process, which enables thorough case assessment through expert review and structured evaluation protocols. Björnsson and Hoofnagle’s^26^ enhanced the database’s utility, by systematically reviewing 671 drugs documented in LiverTox and classifying them based on published idiosyncratic liver injury reports. Their analysis applied RUCAM to specific case categories, establishing a robust causality framework. The LiverTox database represents a significant advance in DILI assessment through its comprehensive approach.^24^ The LiverTox likelihood scoring system provides a standardized framework for categorizing drugs based on the strength of evidence for either direct or idiosyncratic hepatotoxicity, ranging from Category A (definite likelihood with >50 reported cases) to Category E (unlikely to cause liver injury despite extensive use). When not enough data to evaluate liver injury risk are available (this is often the case for newly introduced or infrequently used drugs), the potential of these molecules for hepatotoxicity is classified as “unknown.”

A significant challenge in DILI analysis has been the lack of standardization in risk assessment across the scientific literature. In 2016, Chen and colleagues^27^ addressed this issue by developing an annotation scheme for weighing evidence of causality, to overcome inherent limitations in drug labeling information and improve the accuracy of DILI annotation. They compiled and extended the LiverTox data set by further refining the classification scheme and including additional drugs and implemented DILIrank 1.0, a verification process that assesses whether drugs have been confirmed to cause DILI using publicly available sources such as the Spanish DILI Registry, the Swedish Adverse Drug Reactions Advisory Committee Database, the Drug-Induced Liver Injury Network (DILIN), and the LiverTox database itself. This process relies on reported data, that is, already existing knowledge, allowing the annotations to remain current. The system categorizes 1036 FDA-approved drugs, including those later withdrawn from the market, into four distinct DILI risk classes, offering a standardized framework for evaluating the DILI potential. The DILIrank classification scheme categorizes drugs into four risk levels based on documented evidence of liver injury, regulatory actions, and published causality assessments, providing a standard framework for human DILI risk stratification.^27^ Against this backdrop, DILIrank 2.0 ^28^ has recently provided a critically needed update by revisiting the original annotations and incorporating newly reported DILI cases together with 300 additional nonbiologic drugs approved between 2010 and 2021. The updated data set covers 1336 compounds and captures shifts in the DILI liability landscape over eight decades of FDA approvals, with an expanded representation of high-risk classes such as antineoplastic and immunosuppressant agents. Importantly, DILIrank 2.0 was explicitly designed not only as a reference list for human DILI risk but also as a benchmark for evaluating new approach methods including in vitro assays, in silico models, and other alternative test strategies, by providing a common, regulatory-grade ground truth for performance comparison.^28^

Complementary to DILIrank, DILIst^29^ provides a harmonized binary classification of human hepatotoxicity that is particularly convenient for modeling. Developed by Thakkar et al., this data set integrates annotations from multiple earlier sources—including the Greene,^20^ Suzuki,^30^ and Zhu cohorts^23^ as well as Björnsson and Hoofnagle^26^ derived lists—and maps them onto a simple DILI-positive versus DILI-negative end point for 1279 drugs (768 positives, 511 negatives).^29^ By cross-linking each compound to PubChem, DrugBank, and KEGG, DILIst couples curated labels with rich chemical and pharmacological information, making it a practical benchmark for QSAR and machine learning models that require large, structurally diverse data sets with consistent binary DILI annotations.

Table 1 summarizes the key characteristics of major DILI annotation data sets, illustrating the evolution of data collection approaches from early efforts with limited scope to comprehensive resources integrating multiple data sources and standardized classification schemes.

DILI classification presents several challenges, and treating it as a binary problem often oversimplifies the underlying complexity. In addition, the use of multiple, evolving classification schemes has introduced inconsistencies across the studies. Other difficulties include accounting for dose dependency and interindividual variability, such as genetic or physiological factors. These issues have prompted continued efforts to improve data curation practices and causality assessment methods in DILI research. Given that accurate classification is already difficult, developing predictive models based on descriptive data is even more complex. The limitations discussed above further compromise the reliability and performance of such models.

Beyond their descriptive value, the data sets summarized in Table 1 outline what current DILI prediction models can, and cannot, reasonably achieve, providing a critical framework for expectation management. In our view, four aspects are particularly relevant for informing the design. First, annotation consistency is limited: early collections (e.g., Cruz-Monteagudo,^17^ Xu,^19^ Greene^20^) rely largely on case reports and labels with heterogeneous causality assessment, whereas DILIrank^28^ and DILIst^29^ introduce more standardized but still evolving schemes. As a result, different models may in fact be learning different, and sometimes conflicting, definitions of “DILI-positive”. Second, the quality of negative labels is often uncertain because the absence of reported hepatotoxicity does not guarantee true safety, especially for recently approved or rarely used drugs.

Additionally, small data sets could provide limited chemical space coverage, restricting model applicability to novel early scaffolds outside the training set.^31^

This uncertainty complicates risk–benefit decisions when a model predicts low DILI risk for a new scaffold. Third, almost all data sets lack explicit dose and exposure information. They therefore encode molecular hazard rather than quantitative risk, which is problematic because many clinically important DILI liabilities emerge only at higher systemic or hepatic exposures.^32^ Fourth, mechanistic annotations (e.g., bioactivation, bile salt export pump (BSEP) inhibition, mitochondrial toxicity, immune mediation) are sparse, limiting the development of models that can offer mechanistically interpretable predictions.

Recent models incorporate dose-dependency by combining pharmacokinetic (PK) with in vitro/mechanistic data for quantitative risk. Chen et al. pioneered this using daily dose (>100 mg), log *P* (≥3), and RM potential across 354 FDA drugs, yielding severity scores correlated with clinical outcomes.^33^ ToxPredictor^34^ integrates multi-concentration RNA-seq data from primary human hepatocytes with clinical *C*_max_ values to generate exposure-adjusted DILI probabilities and safety margins (e.g., >80-fold), achieving 88% sensitivity and 100% specificity in validation. The model captures transcriptional signatures of mitochondrial dysfunction, oxidative stress, immune activation, and ferroptosis. These pathways are implicated in approximately 44% of idiosyncratic DILI cases, including those associated with genetic susceptibility.^34^ Similarly, Geci et al.^35^ demonstrated that combining in vitro toxicity data (cytotoxicity, mitochondrial toxicity, BSEP inhibition) with pharmacokinetic information enables high DILI predictivity (AUC 0.96). Hybrid workflows integrating structural features with pharmacokinetic and in vitro data thus enable quantitative assessment of DILI risk. An additional, often overlooked complication is that DILI end points differ substantially across data sets. Some resources focus on any reported liver signal, others on severe clinical DILI, and others on idiosyncratic cases with stringent causality assessment. Therefore, models trained on different data sets are effectively optimizing for different definitions of DILI and are not directly interchangeable.

Taken together, these limitations mean that DILI models trained on these data sets are best used as early warning tools that flag compounds requiring closer mechanistic analysis, rather than as independent gatekeepers deciding which molecules should or should not be progressed. For robust decision-making, medicinal chemists should (i) select models trained on a clearly defined, homogeneous end point relevant to their project needs and (ii) use consensus predictions across complementary models when evaluating new chemical matter. When multiple independent models, each aligned with different DILI end points, converge on high risk for a given scaffold, this provides a strong signal to modify that series; conversely, disagreement between models should be taken as uncertainty rather than reassurance of safety. When designing new analogues, medicinal chemists should look for consensus across models trained on different DILI data sets. Agreement on “low risk” across end point-specific models provides confidence to expand that chemical series, with disagreement signal uncertainty requiring experimental follow-up.

### DILI Prediction Models: Evolution of Computational Methods

Experimental DILI risk assessment has relied on cell lines and, mostly, on animal models.^36^ However, obtaining data from animal models, while widely adopted as a strategy, is time-consuming and labor-intensive and, most importantly, does not necessarily provide insights into human toxicity responses.^37^ This is likely due to substantial differences in drug metabolism and disposition between animals and humans. These differences arise partly from species-specific expression of cytochrome P450 enzymes (CYPs) and other key drug-metabolizing enzymes, as well as genetic variability within the human population.^38^ While in vitro approaches offer a practical venue, they have their own limitations, such as the challenge of selecting appropriate assays and translating assay concentrations into in vivo blood levels associated with hepatotoxic risk.^39^

These significant limitations are driving the field toward the systematic adoption of computational alternatives. In silico methods, particularly ML and DL approaches, have been extensively used for DILI predictions. In this perspective, we will focus mainly on DILI risk prediction models based on molecular structures or molecular descriptors. These approaches can rapidly screen many compounds, potentially enabling earlier identification of hepatotoxicity risks during drug development at a lower cost. The success of computational DILI prediction fundamentally depends on two critical elements: how molecular structures are represented in computer-interpretable formats and which algorithms can best identify patterns within these representations that correlate with hepatotoxic outcomes. The evolution of both elements has been directly influenced by the size, quality, and annotation schemes of the DILI data sets described above.

For computational predictions to be effective, molecules must first be translated into computer-interpretable formats. Four main approaches have emerged for representing molecular structures in DILI prediction models: SMILES strings, which represent molecules as linear text notation;^25^ molecular descriptors,^40^ which provide numerical values for physicochemical properties; molecular fingerprints,^41^ which encode the presence of specific structural features as binary vectors;^42^ and molecular graphs, which preserve complete structural information by representing atoms as nodes and bonds as edges.^43^ Importantly, the choice of molecular representation strongly influences the types of algorithms that can be used. Traditional ML models typically require precomputed features such as descriptors or fingerprints. In contrast, many DL approaches, particularly those based on graph neural networks or sequence models, can operate directly on structured representations such as molecular graphs. However, DL models often still require preprocessing steps—such as tokenization, embedding, or feature encoding—to convert raw structural data into formats suitable for neural network architectures. Early computational efforts employed conventional ML techniques such as *k*-nearest neighbor Bayesian modeling, random forest (RF), support vector machine, and extreme gradient boosting algorithms.^44^ These methods yielded encouraging results in hepatotoxicity prediction by establishing correlations between structural characteristics and biological activities based on known activity data sets using various statistical algorithms, but the field has subsequently evolved toward DL approaches, with several studies exploring neural networks for DILI prediction to capture more complex, nonlinear relationships and handle larger, more diverse data sets.^45^ In parallel with these algorithmic advances, ensemble methods, namely, methods that combine multiple models, have consistently outperformed individual approaches.^46^ At the same time, interpretability techniques has become increasingly important for gaining mechanistic insights into DILI.^47,48,49,50,52,53,59,64,65,66^

Table 2 summarizes the evolution of computational DILI prediction methods based primarily on molecular structures or molecular descriptors, highlighting the progression from early ML approaches to current state-of-the-art ensemble and DL models, reflecting both methodological innovations and the availability of larger, higher-quality data sets.

**Table 2:**
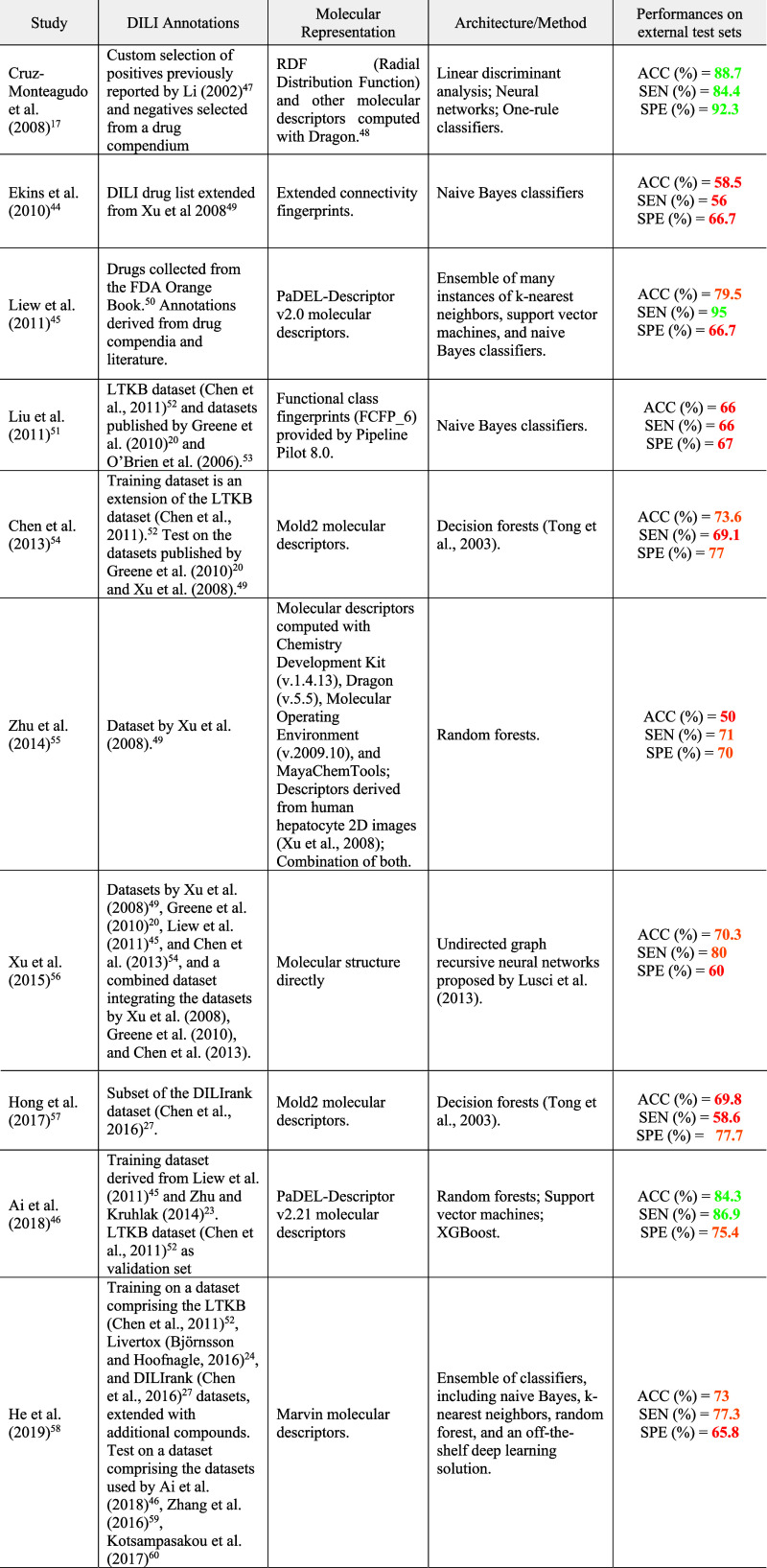

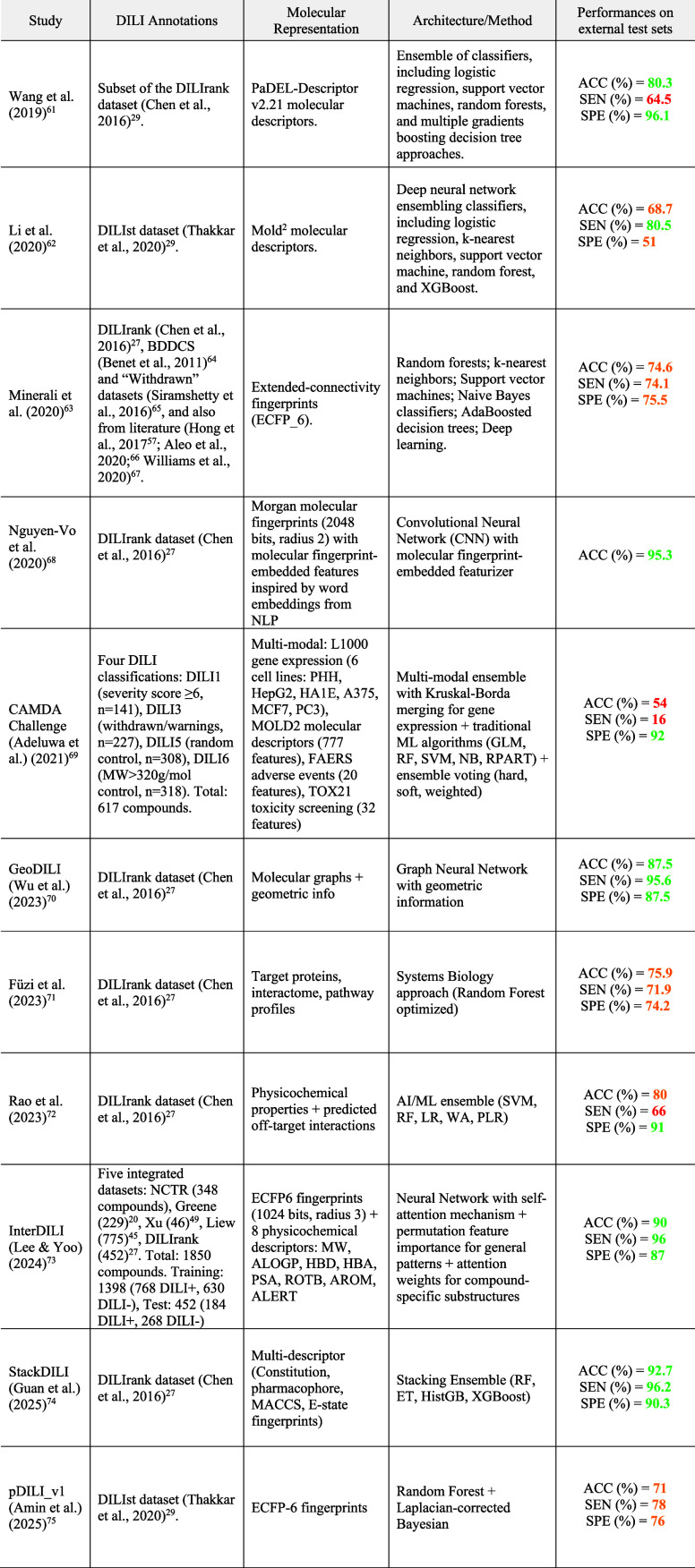
Evolution of Computational DILI Prediction Methods Based on Molecular Structures^a^

a Chronological summary of computational approaches for DILI prediction, highlighting the temporal progression from early ML methods to current state-of-the-art ensemble and DL models. The table presents studies in chronological order and includes study details, DILI annotation sources, molecular representations used, architectural/methodological approaches, and reported performance metrics (e.g., AUROC (area under the receiver operating characteristic curve), ACC (accuracy), SEN (sensitivity), and SPE (specificity)) on external independent test sets, demonstrating the field’s systematic advancement over time from simple descriptor-based models to sophisticated multi-modal ensemble systems. *Cell color coding: green > 0.80 (excellent), orange 0.70–0.80 (good), and red < 0.70 (fair).

Notably, while most approaches relied solely on chemical structures, recent ensemble methods have begun integrating additional data types such as off-target interactions, gene expression profiles, and biological pathway information.

Cruz-Monteagudo et al.^17^ pioneered this field using a small data set of 74 drugs annotated with molecular descriptors computed by Dragon software,^76^ systematically comparing linear discriminant analysis, neural networks, and one-rule classifiers to demonstrate that meaningful structure-toxicity patterns could be identified despite data limitations. Expanding on this earlier work, Ekins et al.^44^ combined a molecular description-based extended-connectivity fingerprints (ECFPs)^77^ with Naive Bayes classifiers, establishing fingerprint-based approaches as a viable alternative to descriptor-based methods and influencing the subsequent widespread adoption of fingerprint representations. The work by Liew et al.^45^ combined multiple classifiers (*k*-nearest neighbors, support vector machines, and naive Bayes) with PaDEL descriptors to analyze a data set comprising 1274 drugs, demonstrating that ensemble approaches could leverage the complementary strengths of different algorithms, a principle that continues to dominate current approaches. Liu et al.^51^ expanded the methodological foundation by applying naive Bayes classifiers to functional class fingerprints (FCFP_6)^77^ across multiple data sets, demonstrating the importance of cross-data set validation and showing how different fingerprint types could capture complementary structural information. Chen et al.^54^ improved traditional ML approaches by applying decision forests to Mold2 molecular descriptors, demonstrating the effectiveness of tree-based methods for DILI prediction through extensive cross-data set validation. In parallel, Zhu et al.^55^ combined multiple descriptor types with human hepatocyte imaging data by using RF algorithms. This represented one of the first efforts to integrate heterogeneous data sources, anticipating later developments in multi-modal modeling. Eventually, Xu et al.^56^ introduced DL architectures by applying undirected graph recursive neural networks to molecular structures. This work demonstrated that DL could process raw structural data without precomputed features, highlighting the potential for end-to-end molecular learning approaches.

The availability of standardized DILI annotation data sets, particularly DILIrank, enabled researchers to focus on algorithmic advancement rather than data curation challenges. Hong et al.^57^ demonstrated how standardized data sets enabled consistent modeling approaches across research groups: their systematic comparison of binary versus 3-class DILI classification revealed that multiclass models provided higher resolution for estimating DILI risk and improved capability to differentiate most-DILI drugs from no-DILI drugs, challenging the field’s predominant focus on binary classification approaches. Increasingly, ensemble methodologies emerged, as highlighted by Ai et al.,^46^ who systematically compared RFs, SVMs, and eXtreme gradient boosting (XGBoost). XGBoost was particularly effective while setting new standards for algorithmic evaluation. Their ensemble approach, combining three ML algorithms with 12 molecular fingerprints on a data set of 1241 diverse compounds, demonstrated the power of ensemble methodology by achieving an accuracy of 84.3% and an AUC of 0.904 on external validation, significantly outperforming individual algorithms. Beyond performance improvements, their work identified several substructures related to DILI and was made accessible through a web server, highlighting the increasing focus on both model complexity and practical applicability.

Similarly, He et al.^58^ developed comprehensive QSAR approaches to predict DILI using Marvins molecular descriptors. Their ensemble method, which averaged probabilities from eight classifiers on a large-scale data set of 1254 compounds, achieved strong performance (accuracy: 73%, sensitivity: 77.3%, AUC: 0.859) and significantly outperformed prior studies through rigorous validation on multiple external test sets. Wang et al.^61^ created ensemble architectures presenting a two-class ensemble classifier model for predicting DILI, with 2D molecular descriptors and fingerprints on a data set of 450 compounds. The purpose of this study was to investigate which are the key molecular features associated with DILI risk and, then, to obtain a reliable ensemble model implementing them.

Li et al.^62^ leveraged the DILIst data set to develop DeepDILI. This DL-powered model combines model-level representations generated by conventional ML algorithms with a DL framework based on Mold2 descriptors. In their study, DL methods achieved a 25.86% improvement in the Matthews correlation coefficient compared to traditional descriptor-based approaches and outperforming conventional ML algorithms and ensemble methods, highlighting the potential of DL architectures for DILI prediction.^62^ Minerali et al.^63^ compared several algorithms using ECFP_6 across multiple data sets, systematically evaluating diverse approaches and establishing RF as consistently effective. Their evaluation of Bayesian ML models alongside k-nearest neighbors, support vector classification, AdaBoosted decision trees, and DL methods revealed comparable performance across algorithms. Importantly, in this work, data sets from industry sources (Pfizer, AstraZeneca) were integrated with FDA data to generate MegaTox, a tool for DILI prediction, which suggests how valuable industry–academia collaborations could be.^63^ Nguyen-Vo et al.^68^ successfully used convolutional neural networks to learn from molecular fingerprints, demonstrating that DL architectures could extract complex patterns from binary molecular representations.

### Model Explainability for DILI

While accurately predicting DILI risk is important, from a medicinal chemistry perspective, it is equally critical to understand the underlying reasons a molecule is flagged as high risk. Identifying which molecular substructures and physicochemical properties are most commonly associated with DILI enables the development of structural alerts (SAs) and guides the design of safer compounds. In GeoDILI, molecules are represented as graphs while explicitly incorporating geometric information such as bond angles.^70^ A gradient-based algorithm then identifies DILI-related substructures, providing model interpretability alongside improved accuracy.^70^ On the other hand, the InterDILI study demonstrated the effectiveness of combining representations based on ECFP with selected physicochemical properties like lipophilicity, number of aromatic rings, number of rotatable bonds, and others to achieve robust predictions.^73^ Lipophilicity and aromatic ring count were consistently identified across studies as the properties most strongly associated with DILI risk; these findings align with known mechanisms of hepatotoxicity.^78^ In terms of structural patterns, InterDILI utilizes permutation feature importance and attention mechanisms to recognize both general and specific structural patterns contributing to DILI risk.^73^ In ML, attention is a function that allows the model to focus on the most relevant parts of a molecule, analogous to how a chemist might prioritize certain functional groups when assessing potential toxicity.^73^

The models summarized in Table 2 illustrate a clear progression from early descriptor-based classifiers to sophisticated ensemble and DL approaches, with external test accuracies typically spanning 0.70–0.85. While early models often functioned as black boxes, generating DILI probabilities without mechanistic explanation, interpretability has emerged as a key requirement in more modern implementations. For medicinal chemists, this evolution suggests a practical workflow: initial rapid screening with high-throughput web-accessible platforms, mechanistic deconvolution using interpretable models like GeoDILI and InterDILI, and prospective risk quantification via end point-specific hybrid approaches.^70,73^ Future DILI assessment will likely combine structural predictions with end point-specific in vitro readouts—cytotoxicity (hepatocellular injury), mitochondrial toxicity (bioenergetic failure), BSEP inhibition—normalized by pharmacokinetic exposure (*C*_max_ ratios),^79^ alongside genetic data revealing susceptibility mechanisms. These diverse mechanistic inputs enable matching experimental readouts to specific liability hypotheses while providing rich training features for next-generation machine-learning models.

Studies focused on interpretability have converged on the identification of several molecular determinants of hepatotoxicity risk, such as aniline derivatives, *N*-benzylformamide, hydrazine-containing compounds, chlorobenzenes, and sulfonamides. Table 3 presents a comprehensive analysis of SAs associated with DILI risk, showing their prevalence in DILI-positive versus DILI-negative compounds in the DILIst data set.^29^ Representative drug examples and the enrichment factor (EF), with an EF of >1 indicating that the substructures identified are over-represented among DILI-positive compared to what would be expected by chance within the data set, are reported.

**Table 3:**
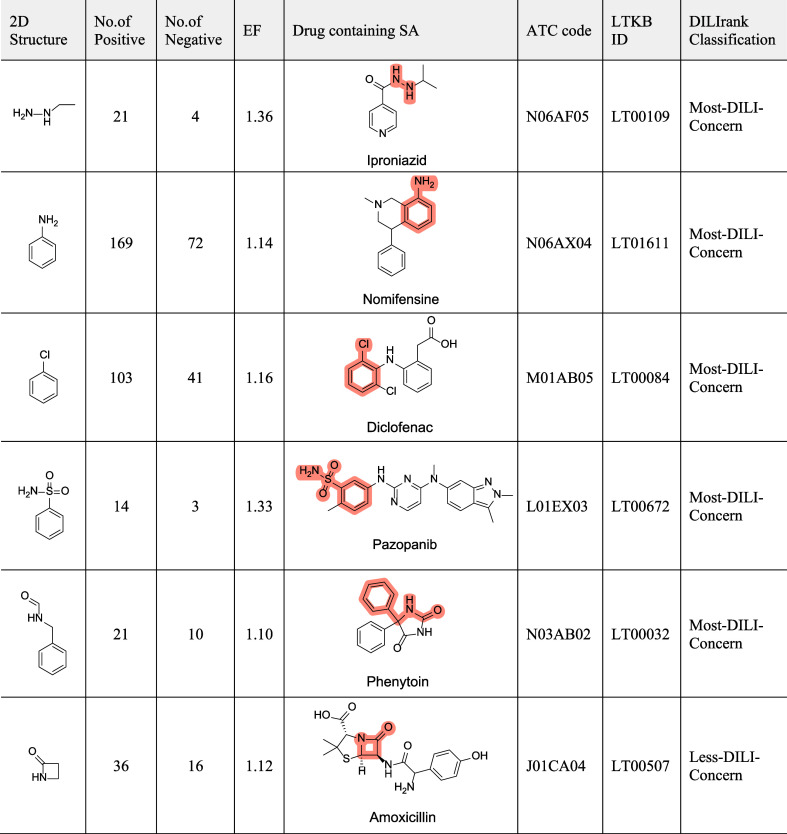
SAs for DILI with Drug Examples^a^

a Visual representation of key SAs associated with DILI risk, including their 2D chemical structures, number of positive and negative compounds in the DILIst data set, enrichment factors, representative drugs containing these moieties, their Anatomical Therapeutic Chemical classification code, Liver Toxicity Knowledge Base IDs, and DILIrank classifications. EF is calculated as (most_DILI_with_alert/total_with_alert)/(total_most_DILI/data set_size). EF > 1 is set as a threshold to identify substructures over-represented in Most-DILI compounds. Higher EF indicates better enrichment of true positives in top rankings.

The hydrazine SA is particularly concerning, appearing in 21 DILI-positive compounds in the DILIst data set as it is a known human carcinogen capable of causing hepatic necrosis, leading to acute liver failure.^80^ The aniline structural motif shows considerable prevalence, with 169 DILI-positive compounds and an EF of 1.14. The hepatotoxic mechanism of aniline has been extensively studied in primary cultured hepatocytes, where exposure leads to dose-dependent generation of ROS, depletion of antioxidant defenses (glutathione, superoxide dismutase, and catalase), mitochondrial dysfunction with loss of membrane potential, DNA damage, and ultimately apoptotic cell death in hepatocyte models.^81^ Nevertheless, SAs should be interpreted primarily as empirical indicators of increased DILI liability rather than direct mechanistic determinants of toxicity. This distinction is exemplified by Nomifensine, a dopamine reuptake inhibitor antidepressant, classified as Most-DILI-Concern in the DILIrank system and withdrawn from the market in 1992.^82^ Although Nomifensine contains an aniline-related motif, its hepatotoxicity has also been hypothesized to arise from oxidative bioactivation pathways, leading to the formation of highly reactive quaternary dihydroisoquinolinium and isoquinolinium intermediates.^83^ This observation underscores an important limitation of many current ML-based DILI prediction models and SA approaches. While these methods successfully identify empirical associations between substructures and hepatotoxicity risks, they may fail to fully capture metabolic activation pathways and transient reactive intermediates that arise during biotransformation. Consequently, SAs should not necessarily be interpreted as direct mechanistic toxicophores but rather as statistically enriched molecular features associated with increased DILI liability within specific chemical and metabolic contexts. The chlorobenzene moiety emerges as another particularly significant group, present in 103 DILI-positive compounds with an EF of 1.16. Indeed, it is known to be metabolized in the liver by cytochrome P450 enzymes to form epoxides, which in turn bind to proteins, DNA, and RNA, thereby contributing to toxicity.^84^ Notable examples include diclofenac, a widely used nonsteroidal anti-inflammatory drug classified as Most-DILI-Concern, ultimately highlighting how even commonly prescribed medications can carry hepatotoxic risk. Sulfonamide-containing compounds represent another critical structural class, with 14 DILI-positive cases showing the highest EF value of 1.33. Sulfonamides are among the most frequently implicated drug classes in IDILI, often presenting with immune-mediated hypersensitivity. Pazopanib, an example reported in Table 3, is a potent and selective tyrosine kinase inhibitor approved for the treatment of advanced renal cell carcinoma. Consistent with the DILI alert, clinical trials of pazopanib showed severe and fatal hepatotoxicity in treated patients.^82^

The N-benzylformamide structural motif, present in 21 DILI-positive compounds, represents another significant hepatotoxicity alert with phenytoin serving as a prominent clinical example. Phenytoin is an aromatic anticonvulsant with well-documented hepatotoxicity, classified as Most-DILI-Concern, and it causes severe liver injury in 1 per 10,000 to 1 per 50,000 exposures as part of an idiosyncratic reaction through formation of reactive arene oxide metabolites that trigger oxidative injury with immune-mediated responses.^85^

Finally, the 2-azetidinone ring system (β-lactam structure), exemplified by amoxicillin in the table, appears in 36 DILI-positive compounds with a moderate EF of 1.12. Amoxicillin is classified as Less-DILI-Concern in the DILIrank system, which aligns with the general clinical observation that amoxicillin and other aminopenicillins have been linked with idiosyncratic liver injury, but only rarely and in isolated case reports.^67^ Amoxicillin causes liver injury in 0.3 of 10,000 prescriptions when administered alone,^86^ demonstrating its favorable hepatic safety profile compared to the combination with clavulanate. This reflects the well-established principle that β-lactam antibiotics generally have little hepatotoxic potential, with liver injury being extremely rare for most penicillins and cephalosporins.^87,88^ The β-lactam ring, recognized as a DILI SA in the literature,^70^ allows for further considerations. The presence of a potentially hepatotoxic substructure does not necessarily translate to significant DILI risk, as SAs flag substructures without considering their broader chemical context. For this reason, manual annotation, or the use of next-generation transformer-based learning architectures with enhanced context awareness, may be necessary to identify true DILI-phores, defined as substructures associated with DILI risk only when present in specific chemical environments.

Despite these contextual limitations, these SAs represent important molecular determinants of hepatotoxicity risk, each contributing through distinct mechanistic pathways, ranging from RM formation (chlorobenzenes, anilines) to immune-mediated hypersensitivity (sulfonamides) and mitochondrial dysfunction (β-lactams). The varying enrichment factors (from 1.10 to 1.36) reflect the complex and multifactorial nature of DILI, where structural features interact with pharmacokinetic properties, dosing regimens, and patient-specific factors to determine overall hepatotoxic potential. Although no single alert is strongly predictive in isolation, high-prevalence motifs (e.g., aniline, *n* = 169; EF = 1.14) provide actionable signals when interpreted alongside mechanistic rationale and cross-model consensus, thereby guiding structure-based risk mitigation during molecular design. Importantly, these SAs can serve as valuable input features for ML-based DILI prediction models, providing mechanistically informed descriptors that capture known DILI-phores and complement traditional physicochemical properties.

The combination of geometric information, SAs, and physicochemical properties represents a significant advance over traditional QSAR approaches, offering both improved predictive power and mechanistic insights.^89^ Amin et al. introduced pDILI_v1,^75^ a Web-based ML tool that addresses a critical gap by systematically exploring the chemical space associated with hepatotoxicity.^75^ This approach integrates chemical space analysis with molecular fingerprints to enhance DILI prediction, focusing on three key objectives: exploring the chemical space and scaffold diversity associated with DILI, identifying through fragment-based approaches SAs that influence DILI risk, and developing supervised ML models to predict DILI risk while elucidating the structural significance of molecular fingerprints.

The pDILI_v1 framework utilizes a Laplacian-corrected Bayesian model to analyze ECFP-6 fingerprints, identifying critical substructural features such as aromatic acids, furans, and substituted triethylamines as hepatotoxicity markers.^75^ Furthermore, through partial dependence plots, the model attempts to explain how specific descriptors like SlogP (Logarithm of the Octanol–Water Partition Coefficient) and GATS 2d (Geary Autocorrelation Descriptor, Lag 2, Distance-Based) a 2D molecular descriptor based on the Geary autocorrelation function contribute to hepatotoxicity predictions.^75^ For example, the analysis demonstrates that compounds with slog *P* values higher than 3.5 combined with high GATS 2d values exhibit increased DILI risk, providing actionable insights for medicinal chemists during lead optimization.^75^ Building upon these advances, Guan et al. have most recently developed StackDILI,^74^ which, unlike previous models that relied on singular molecular representations or individual algorithms, improves prediction accuracy and interpretability by integrating multiple molecular descriptors to extract comprehensive structural and physicochemical information, MACCS, and E-state (electrontopological state) fingerprints.^90^ This multi-descriptor approach captures a broader range of molecular properties relevant to hepatotoxicity prediction than single-representation models. StackDILI implements a genetic algorithm for feature selection and optimization. This evolutionary approach identifies the most relevant features from a high-dimensional feature space, addressing limitations of previous models that either used all available features (introducing noise) or relied on manually selected features (potentially missing important patterns). The selected features are then processed through a stacking ensemble architecture that leverages multiple tree-ML models, including RF, extremely randomized trees (ET), histogram-based gradient boosting (HistGB), and XGBoost.^74^ This multi-model approach allows each algorithm to contribute its unique strengths, with a final layer combining these insights for superior performance.

When evaluated on the DILIrank test set, StackDILI demonstrated good performance, achieving an accuracy of 0.93, a sensitivity of 0.96, and a specificity of 0.90, significantly outperforming previous approaches. Beyond its predictive capabilities, StackDILI provides substantial interpretability insights through SHapley additive exPlanations (SHAP) values,^91^ revealing that MACCS fingerprints contribute approximately 60% of the predictive power, followed by pharmacophore features at 23%. Specifically, the model identified MACCS135 (representing nitrogen atoms not bonded within aromatic systems but connected to two other atoms) and MACCS94 (describing heteroatom-nitrogen connections) as particularly strong indicators of the DILI risk.

Both pDILI_v1 ^75^ and StackDILI^74^ exemplify the current trend toward providing accessible Web-based interfaces, representing a significant step forward in making sophisticated DILI prediction tools readily available to medicinal chemists and facilitating the integration of hepatotoxicity assessment early in the drug development process.

### Integration of Multiple Data Types in DILI Prediction Models

The high variability in individual responses to drugs, largely influenced by genetic factors, highlights the need for more personalized methods in DILI prediction. Tailoring predictions to individual characteristics, i.e., genetic variations, would not simply refine existing models; it could represent a fundamental step forward in capturing the complexity of DILI, particularly in its idiosyncratic forms.^92,93^ To this aim, integrated approaches, which combine various data sources such as molecular structures, gene expression profiles, clinical reports, and toxicology screening results, have been proposed. A first attempt at multi-modal integration was undertaken through the CAMDA Drug Safety Challenge, which integrated gene expression data from multiple cell lines (PHH, HepG2, HA1E, A375, MCF7, PC3), MOLD2 molecular descriptors encoding 2D chemical structures, post-marketing drug adverse event information from the FDA Adverse Event Reporting System (FAERS), and high-throughput liver toxicity screening results from TOX21.^69^ To do that, the CAMDA Drug Safety Challenge employed ensemble approaches for combining gene expression data with traditional ML algorithms and ensemble voting strategies.^69^ However, the CAMDA Challenge achieved only modest balanced accuracies, ranging from 0.54 to 0.60 for clinically relevant DILI subtypes. Individual data modalities showed similarly limited performance: FAERS-based models achieved accuracies of 0.56–0.67, while MOLD2 and TOX21-based approaches showed comparable performance levels.^69^

On the basis of this, we can argue that the limited success of data integration may be attributed to several factors: the technical complexity of combining heterogeneous data types, inconsistent data quality across sources, insufficient understanding of relationships between different data types and DILI mechanisms, reduced sample sizes resulting from data intersection, and the inherent complexity of DILI mechanisms that may exceed the capabilities of current data types.

In another study, Füzi et al.^71^ explored a system biology-based approach that combined three levels of biological information: target proteins, protein–protein interactions (interactome), and biological pathway profiles.^71^ Using RF optimization on the DILIrank data set, this approach achieved an accuracy of 0.77 while identifying important biological processes involved in DILI, including cytochrome P450 enzymes, heme degradation, and biological oxidation pathways. Their methodology used compounds’ annotations to tissue-specific biological targets, interactome networks, and pathways as molecular descriptors, demonstrating that systems biology-based descriptors could effectively capture hepatotoxicity-relevant information.^71^ Interestingly, when the authors attempted to combine their system biology descriptors with structural information via traditional Morgan fingerprints, they observed no significant improvement in model performance. This finding suggests that the biological information captured through target proteins, interactome, and pathway analysis may capture toxicity-relevant information that overlaps substantially with structural descriptors, highlighting potential redundancies between different data modalities rather than true complementarity.^71^

In contrast, Rao et al.^72^ developed an AI/ML ensemble approach that achieved a satisfactory performance (AUC: 0.88, sensitivity: 0.73, specificity: 0.90) by combining physicochemical properties with predicted off-target interactions.^72^ Their model integrated key physicochemical descriptors (fsp3, log S, basicity, reactive functional groups, predicted metabolites) with 43 previously identified off-target interactions discriminating Most-DILI (M-DILI), including PTGS1 (COX1), PTGS2 (CoX2), CYP2C9, RET, ABCC4 (bile efflux), SLC22A12, PPARγ, RXRA, AKR1C3, MGLL, and AR, thus providing valuable mechanistic insights and demonstrating the critical importance of off-target binding in DILI mechanisms.^72^

A representative example is PPARγ: hepatic PPARγ mRNA expression was reported to be increased by 112% and 188% in obese patients with steatosis and steatohepatitis, respectively, relative to normal liver tissue. These findings suggest a mechanistic link between elevated PPARγ signaling and the progression of hepatic steatosis.^94^ Another example is RET Kinase: selpercatinib and pralsetinib, potent and selective RET inhibitors, have been associated with elevated liver enzymes in a substantial proportion of treated patients.^95,96^ Preclinical studies implicate the Wnt signaling pathway, an established crosstalk partner of receptor tyrosine kinases such as RET, in the maintenance of hepatic homeostasis. These observations support the hypothesis that perturbation of the RET/Wnt axis may contribute to the clinical DILI observed with selpercatinib and pralsetinib.^97,98^ Furthermore, the structurally distinct scaffolds of these two compounds suggest that the observed DILI is more plausibly due to the modulation of a shared biological target rather than to scaffold-specific chemical features.

The evolution of DILI prediction models demonstrates a clear progression from simple structural approaches to sophisticated ensemble and DL strategies. While comprehensive multi-modal integration remains challenging, targeted combinations of complementary information, particularly physicochemical properties with biological interactions, have proven to be more successful than broad data integration approaches. These computational approaches have become valuable tools for early-stage drug development, offering both predictive capability and mechanistic insights while becoming increasingly accessible through web-based platforms. Nonetheless, the reported examples provide us with evidence that such multi-modal approaches require careful selection of truly complementary data types and discourage the mere combination of all available information.

### Final Thoughts

DILI is often simplified as a classification problem.^46^ However, simple labels do not provide information on important factors such as dose dependency or affected patient population, and consequently, the practical applicability of such models remains limited. Although it is challenging to obtain precise quantitative data about DILI, existing DILI annotation data sets provide varying levels of evidence for DILI cases. Undeniably, the quality of these data plays a crucial role in the development of reliable prediction models. Kotsampasakou et al.^60^ emphasized that a smaller data set with high-quality, well-verified DILI cases leads to better predictive models than larger data sets with less reliable information. Besides the intrinsic quality of the data, data set imbalance between DILI-positive and DILI-negative cases can also skew model predictions. The over-representation of nontoxic compounds can lead to biased risk assessments, potentially compromising the reliability of predictive models. Similarly, inconsistent classification schemes across different data sets may lead to conflicting annotations for the same compounds, making it difficult to compare results across studies. Moreover, human hepatotoxicity data are notoriously difficult to collect and are often prone to mislabeling. This challenge arises from several key factors: (i) limited number of case reports and the voluntary nature of adverse event reporting, which leads to underreporting and inconsistent data quality; (ii) restricted access to proprietary and post-marketing human toxicity data, particularly from pharmaceutical companies; and, last, (iii) confounding variables, such as polypharmacy and supplement use, which complicate causal inference, making it difficult to attribute hepatotoxicity to a specific compound.^60^

Regarding DILI prediction, most current models rely predominantly on chemical structure data, missing the opportunity to integrate other relevant biological and clinical factors and address the IDILI complexity. On the other hand, combining structural information with other data proved difficult.

To address these limitations, several improvements can be implemented to enhance the quality and utility of DILI annotation data sets. A fundamental step is to develop standardized classification criteria with clear, quantitative severity scales and unified scoring systems. This standardization should include temporal aspects of DILI development and create consistency across different data sets. Documentation needs to become more comprehensive, incorporating detailed annotations of DILI mechanisms, patient-specific factors, drug–drug interactions, and the timing and progression of liver injuries. In this context, extended and continuously curated resources such as the DILIrank 2.0 ^28^ data set offer an important step toward standardized, evidence-based benchmarks for DILI prediction, providing updated classifications for over 1300 FDA-approved drugs based on systematic review of labeling and literature. Beyond curated safety labels, large toxicogenomics repositories such as DrugMatrix,^99^ Open TG-GATEs,^100^ and others now provide rich transcriptomic and histopathological profiles across hundreds of compounds, doses, time points, and tissues, enabling the development of omics-driven models that can capture early mechanistic signals of hepatotoxicity and complement approaches based on the chemical structure. Furthermore, integrating multiple data types, including chemical structures, gene expression profiles, off-target interactions, clinical observations, patient demographics, and pharmacokinetic data, would provide a more complete picture of the DILI risk factors.

The computational evolution from early descriptor-based approaches to sophisticated ensemble and DL architectures demonstrates remarkable algorithmic advancement, yet the modest performance improvements achieved suggest that current limitations transcend algorithmic inadequacy. The CAMDA Challenge^68^ exemplifies this reality, where comprehensive multi-modal integration failed to achieve breakthrough performance despite accessing diverse data types, highlighting that data quality and biological DILI complexity may represent more fundamental barriers than previously anticipated. Additionally, this outcome underscores a critical principle: effective multi-modal integration requires strategic selection of complementary data sources rather than indiscriminate combination of all available information, as data complementarity, not data abundance, drives predictive performance. Moreover, the emergence of interpretable models represents a significant step forward in understanding DILI mechanisms. The identification of key SAs, from hydrazine and aniline derivatives to chlorobenzenes and sulfonamides, provides valuable mechanistic insights and actionable guidance for medicinal-chemistry optimization. However, these interpretability advances still primarily capture associations rather than establish causal relationships, and their predictive utility remains constrained by the inherent limitations of binary classification schemes.

The recent focus on web-accessible platforms and user-friendly interfaces reflects the field’s advance toward practical implementation. Tools like pDILI_v1 ^75^ and StackDILI^74^ demonstrate how sophisticated computational approaches can be made accessible to medicinal chemists, facilitating early-stage toxicity assessment: modifying or avoiding DILI-phores can help prevent late-stage failures due to toxicity.^101^

Looking toward the future, the field must embrace more nuanced approaches that acknowledge DILI’s multifactorial nature. The integration of pharmacogenomics data holds promises for addressing idiosyncratic reactions, while the development of mechanistically informed models that incorporate pathway-specific information could enhance both prediction accuracy and interpretability. A major frontier in DILI prediction lies in the systematic integration of genetic variability into modeling frameworks. As genomic sequencing becomes increasingly embedded in clinical practice, the linkage of DILI case reports with patient-level genetic data (e.g., polymorphisms in drug-metabolizing enzymes) will enable a new generation of hybrid models that incorporate both chemical liability and host susceptibility. Such cross-talk among molecular design, clinical pharmacology, and genomics represents a necessary step toward truly individualized DILI risk assessment.

As therapeutic modalities diversify, DILI prediction must evolve accordingly: future models will need to move beyond small-molecule paradigms toward integrative, modality-spanning frameworks that capture target biology, immune activation, and platform-specific liabilities.

Success will require coordinated efforts across pharmaceutical companies, regulatory agencies, and academic institutions to establish standardized data collection protocols, develop consensus causality assessment frameworks, and implement flexible consensus modeling strategies that allow medicinal chemists to combine complementary tools (e.g., structure-based, omics-based, off-target, and interpretable models) according to specific project needs, ultimately enabling truly predictive models that can guide clinical decision-making and drug development strategies.

## Abbreviations used

ACC: accuracy
ALP: alkaline phosphatase
ALT: alanine aminotransferase
AUC: area under the curve
AUROC: area under the receiver operating characteristic curve
CYP: cytochrome P450
DILI: drug-induced liver injury
DILIN: drug-induced liver injury network
DILIrank: drug-induced liver injury rank
DILIst: drug-induced liver injury severity and toxicity data set
DL: deep learning
ECFP: extended-connectivity fingerprint
FDA: food and drug administration
FCFP: functional-class fingerprint
GSH: glutathione
IDILI: idiosyncratic drug-induced liver injury
InDILI: intrinsic drug-induced liver injury
KEGG: Kyoto Encyclopedia of Genes and Genomes
LTKB: Liver Toxicity Knowledge Base
ML: machine learning
NAMs: new approach methods
QSAR: quantitative structure–activity relationship
RF: random forest
RMs: reactive metabolites
ROS: reactive oxygen species
RUCAM: Roussel Uclaf Causality Assessment Method
SMILES: simplified molecular-input line-entry system
SVM: support vector machine
TBIL: total bilirubin
WHO: World Health Organization
XGBoost: extreme gradient boosting.
